# A commentary on “PI3K‐α/mTOR/BRD4 inhibitor alone or in combination with other anti‐virals blocks replication of SARS‐CoV‐2 and its variants of concern including Delta and Omicron”

**DOI:** 10.1002/ctd2.87

**Published:** 2022-06-10

**Authors:** Sakthijothi Muthu, Sundararajan Venkatesh

**Affiliations:** ^1^ Department of Physiology and Pharmacology School of Medicine, West Virginia University Morgantown West Virginia USA

In their recent article (PI3K‐*α*/mTOR/BRD4 inhibitor alone or in combination with other anti‐virals blocks replication of SARS‐CoV‐2 and its variants of concern including Delta and Omicron), Acharya and co‐authors demonstrated, by using in vitro cell culture models, that SF2523, a small molecule inhibitor of PI3K‐*α*/mTOR/BRD4 can also inhibit SARS‐CoV‐2 replication, including the variants of concerns such as Delta and Omicron.^1^ Further, they showed that SF2523 acts synergistically with two of the currently available anti‐SARS‐CoV‐2 drugs, remdesivir and MU‐UNMC‐2.^1^ Although authors agree that developing vaccines against SARS‐CoV‐2 is a breakthrough and essential to protecting against its infection and transmission, in this study, they emphasize the importance of treating SARS‐CoV‐2 infection as well. This work demonstrates that SF2523 alone or in combination with existing anti‐ SARS‐CoV‐2 drugs can effectively inhibit replication of SARS‐CoV‐2 and its variants in in vitro cell culture models.^1^

One of the recent threats to human health is the outbreak of recent severe acute respiratory syndrome coronavirus 2 (SARS‐CoV‐2) that caused an acute pandemic of coronavirus disease 19 (COVID‐19) worldwide.^2^, ^3^ Though various theories have been put forward on the mechanism(s) of SARS‐CoV‐2 infection and pathologies; investigations are still underway to thoroughly understand how this RNA virus causes such a devastating effect on the host. Studies have predicted that SARS‐CoV‐2 could interact with various host cell proteins and hijack to sustain its active replication.^4^, ^5^ Consistent with this, recent studies have predicted many interactions between SARS‐CoV‐2 and host proteins that are believed to be essential for the SARS‐CoV‐2 lifecycle. One of such proteins includes BRD2, which is shown to interact with the E protein of SARS‐CoV‐2, leading to downregulation of ACE‐2 expression that blocks SARS‐CoV‐2 entry to host cells.^6^ Our recent review also emphasizes that SARS‐CoV‐2 interactions occur at the organelle levels (e.g., mitochondria), and hence mitochondria are also predicted to be emerging as a potential target in COVID‐19 treatment.^7^

Authors believe that since several SARS‐CoV‐2 proteins interact with host proteins to escape from being eliminated, strategies like inhibition of host‐SARS‐CoV‐2 protein interaction could block the entry of SARS‐CoV‐2 and downregulate the cytokine storm by decreasing certain interferon‐stimulated genes.^6^ Therefore, they strongly suggest that targeted therapies against viral‐host protein interaction would not only benefit SARS‐CoV‐2 treatment but also be less impacted by the presence of SARS‐CoV‐2 variants of concerns. One such class of proteins is BRD2/BRD4, bromodomain‐containing proteins, and are master transcriptional regulators. Therefore, they tested SF2523, an inhibitor or BRD4 against SARS‐CoV‐2 infection. Moreover, the dual advantage of using SF2523 is that since BRD2/BRD4 induces pro‐fibrotic genes and promotes lung tissue fibrosis, which is majorly seen in COVID‐19 patients; therefore, inhibition of BRD2/BRD4 may prevent lung fibrosis and promote fast recovery as well. Interestingly, SF2523 also inhibits PI3K‐*α* and mTOR, which are also shown to interact with SARS‐CoV‐2 proteins.^4^ When authors used SF2523 in cell culture in combination with other existing covid‐19 drugs such as Remdesivir and MU‐UNMC2, they found a significant synergistic effect of these combinations against SARS‐CoV‐2, including against Delta, Omicron, the South African and the Scotland variants.^1^ In addition, through NMR studies, authors confirm that other antiviral drugs don't inhibit SF2523 interactions with BRD4, and hence, the potency of SF2523 is not altered. Although this is an exciting and essential finding in the field, one major caveat is that authors mostly employ invitro cell culture models rather than an in‐vivo model. Although the outcome is intriguing, the observed results could change when tested in preclinical animal models or humans. Therefore, we urge authors to follow up on this work to test in in‐vivo models as this outcome could potentially change COVID‐19 treatment strategies and help us prepare for future similar outbreaks.
